# Benchmarking the Photocatalytic Self-Cleaning Activity of Industrial and Experimental Materials with *ISO 27448:2009*

**DOI:** 10.3390/ma16031119

**Published:** 2023-01-28

**Authors:** Hannelore Peeters, Silvia Lenaerts, Sammy W. Verbruggen

**Affiliations:** 1 Sustainable Energy, Air & Water Technology (DuEL), Department of Bioscience Engineering, University of Antwerp, Groenenborgerlaan 171, 2020 Antwerp, Belgium; 2 NANOlab Center of Excellence, University of Antwerp, Groenenborgerlaan 171, 2020 Antwerp, Belgium

**Keywords:** photocatalytic self-cleaning coating, titania thin film, ISO 27448:2009, industrial self-cleaning materials, self-cleaning surfaces

## Abstract

Various industrial surface materials are tested for their photocatalytic self-cleaning activity by performing the ISO 27448:2009 method. The samples are pre-activated by UV irradiation, fouled with oleic acid and irradiated by UV light. The degradation of oleic acid over time is monitored by taking water contact angle measurements using a contact angle goniometer. The foulant, oleic acid, is an organic acid that makes the surface more hydrophobic. The water contact angle will thus decrease over time as the photocatalytic material degrades the oleic acid. In this study, we argue that the use of this method is strongly limited to specific types of surface materials, i.e., only those that are hydrophilic and smooth in nature. For more hydrophobic materials, the difference in the water contact angles of a clean surface and a fouled surface is not measurable. Therefore, the photocatalytic self-cleaning activity cannot be established experimentally. Another type of material that cannot be tested by this standard are rough surfaces. For rough surfaces, the water contact angle cannot be measured accurately using a contact angle goniometer as prescribed by the standard. Because of these limitations, many potentially interesting industrial substrates cannot be evaluated. Smooth samples that were treated with an in-house developed hydrophilic titania thin film (PCT/EP2018/079983) showed a great photocatalytic self-cleaning performance according to the ISO standard. Apart from discussing the pros and cons of the current ISO standard, we also stress how to carefully interpret the results and suggest alternative testing solutions.

## 1. Introduction

Photocatalytic self-cleaning materials no longer only refer to self-cleaning glass [1,2,3]. In the construction sector, self-cleaning tiles, wallpaper, paint, window blinds, concrete and asphalt are being produced as well [4,5,6]. Moreover, self-cleaning coatings are quintessential for solar cells and photovoltaic panels to keep their yield as high as possible [2,7]. Additionally, more and more indoor self-cleaning materials such as self-cleaning paints [8] and fabrics [9,10] are being used. Even photocatalytically self-cleaning membranes for water purification have been made to battle fouling in (waste) water treatment and membrane distillation [11,12]. To put this into numbers, a quick screening of the scientific and patent research is performed. The first photocatalytic self-cleaning applications appeared in the 1990s, eight decades after the photocatalytic degradation reaction was first described [13,14]. The number of scientific papers mentioning photocatalytic self-cleaning surfaces has increased steadily ever since, but it is far outnumbered by the scientific papers on other photocatalytic applications, specifically for water and air treatments [15]. Additionally, the number of patents concerning photocatalytic applications has grown steadily since the 1990s, with a steep increase in annual new patents in 1995–2000 and a levelling off after 2000, possibly indicating the maturation of the technology. Here, the number of photocatalytic water and air treatments heavily outweigh the number of patents on photocatalytic self-cleaning surfaces. On a cumulative basis, self-cleaning surfaces account for about 9% of them, water treatment accounts for 38% of them and air treatment accounts for 53% of the patents for these applications from 1990 to 2007 [15]. For the readers interested in a patent overview, specifically of air treatment patents, we recommend the review by Paz [15], while specific companies and commercial applications are summarised by Mills and Lee [1].

**Figure 1:**
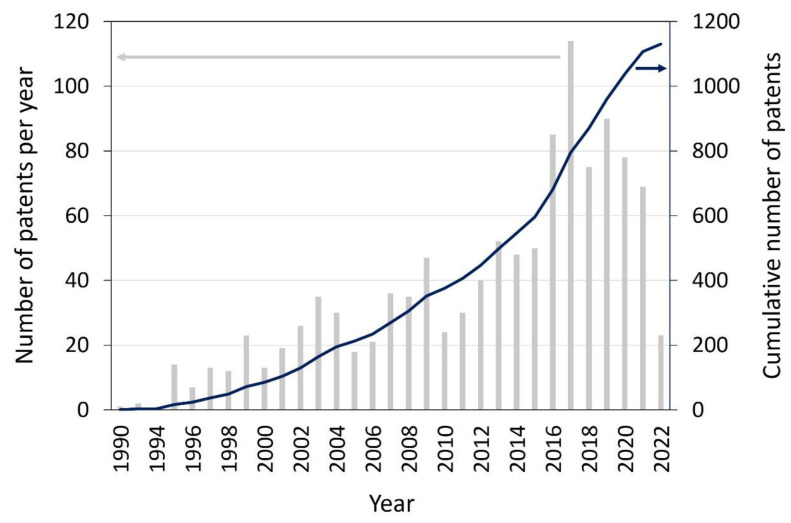
Patents found with Espacenet, EPO’s free patent database (worldwise.espace.com), when we were using the search terms “self-cleaning coating” OR “self-cleaning surface”) AND “photocatalytic” on 10 October 2022.

When we are looking more in detail at photocatalytic self-cleaning materials (‘*photocatalysis’*, ‘*photocatalytic material’* and ‘*self-cleaning’*) using Google Patents search engine, the oldest patents actually do not mention the term ‘*self-cleaning’* explicitly in the patent text. The first patent mentioning any self-cleaning action comprises an electrochemical cell with Ti_4_O_7_ as a photoanode material for the dissociation of water, and it claims the self-cleaning of both the anode and cathode made from a bipolar material by reversing the current (US4422917A, 1981 [16]). With the wide variety of results for these search terms on Google Patents (56,122 in October 2022), of which many appeared to not consider self-cleaning materials, another patent database was used. In the public database Espacenet by the European Patent Office (EPO), 1343 results (in October 2022) were found for the search terms “*self-cleaning*” AND “*photocatalytic*”. The oldest documents treat photocatalytic-binder compositions for various applications including self-cleaning paints and waxes and the removal of contaminants from a fluid stream such as air or water, earliest priority 1990 and 1993, published in 1997 and 1995 respectively (US5616532A, WO9511751A1 [17,18]). After these descriptions for binders, more and more patents for (hydrophilic) coatings covering a variety of materials have been published since 1995 [19]. Selected examples include a washing tank, window glass, cover glass, glass/ceramic/vitroceramic-based substrates, a cover for a solar battery, a lamp/luminaire and a handrail [20,21,22,23,24,25,26]. The mentioned photocatalyst, when it is specified, is in most cases TiO_2_, but also, ZnO and SnO have been used. The more recent patents are more advanced, describing personal apparatus for air disinfection by UV with a photocatalytic self-cleaning coating, including an antiviral effect and a carbon negative self-cleaning inorganic coating [27,28]. The general increasing trend in the number of annually filed patents is not declining thus far, supposing that the 2020–2021 dip can be attributed to the global recession due to the COVID-19 pandemic, as shown in Figure 1. 

The benefits of self-cleaning surfaces speak for themselves. Chemicals/cleaning products, water, energy and labour time are saved. On top of this, the solar energy control can improve, and thus, the energy savings can increase by using self-cleaning surfaces for applications such as photovoltaic panels and smart windows. This is an obvious economic advantage that increases the user’s comfort and benefits the environment [2]. The added benefit of photocatalytic self-cleaning materials is that only light is needed to activate the material. After their activation, the materials break down the fouling agents by generating reactive oxygen species (ROS), on the one hand, and make the photocatalytic surface superhydrophilic/superwettable on the other hand. This way both organic foulants (CH-compounds) can be completely degraded to CO_2_ and H_2_O, while inorganic residues can be easily washed off by the sheeting of water (vide infra) [2]. In order to make the surface photocatalytically active, a photocatalyst is obviously needed. A photocatalyst is typically a semiconductor that is activated by light with sufficient energy to overcome the band gap (*E_g_*). The most commonly used photocatalyst is titanium dioxide (TiO_2_), as mentioned earlier, because this semiconductor is affordable, non-toxic, (photo)chemically stable and easily activated. The incident light wavelength needed to activate the semiconductor corresponds to ≤388 nm for anatase, with a band gap of about 3.2 eV, implying that (near-)UV light is required for the activation. The large band gap enables TiO_2_ to effectively perform many redox reactions, but it is also its largest shortcoming since the solar spectrum at the surface of the Earth only exists for up to 5% of the UV light. Given the importance of an efficient photocatalyst, several strategies to improve the photocatalytic activity of TiO_2_ have been proposed. For the interested reader, we gladly refer the reader to specialised review articles on TiO_2_ photocatalysis (e.g., one by Verbruggen [29]).

Objectively comparing different photocatalytic self-cleaning materials is not an easy task, since the photocatalytic activity depends on various parameters, such as irradiation intensity, catalyst loading, foulant concentration, temperature and relative humidity, etc. Hence, there is a clear need for a standardised protocol. This protocol should homogenise the testing method by setting fixed values for the parameters that influence the photocatalytic efficiency. The International Standard Organisation (ISO), which was founded in London in 1946, is an independent, non-governmental organisation that publishes such international standards [30]. The first ISO standard for photocatalytic materials was published in 2007: ISO 22197-1:2007—Fine ceramics (advanced ceramics, advanced technical ceramics)—Test method for air-purification performance of semiconducting photocatalytic materials—Part 1: Removal of nitric oxide. Soon, other standards for photocatalytic semiconducting materials were published for the removal of other gases (from ISO 22197-2 to -5), for antibacterial activity (ISO 27447:2009), for self-cleaning activity (ISO 27448:2009), for water purification performance (ISO 10676:2010), for photocatalytic activity in aqueous media (ISO 10678:2010), for the UV light source to be used when testing (ISO 10677:2011) and for antifungal, antiviral and antialgal activity (ISO 13125:2013, 18061-1:2014 and 19635:2016 resp.). In recent years, a true increase in the number of standards has taken place with the publishing of seventeen standards in only four years, and there are two more standards under development. The increase in the number of standards takes the use of different light sources and environments into account (e.g., LED lights and indoor lighting environments), as well as newer, safer (revised standards) and more accurate testing methods for different photocatalytic materials (e.g., in situ FTIR spectra analysis with a recirculating air flow photoreactor for building/construction materials). In this article, we will focus on ISO 27448:2009-Fine ceramics (advanced ceramics, advanced technical ceramics)-Test method for self-cleaning performance of semiconducting photocatalytic materials-Measurement of water contact angle. This standard was first published in 2009, and it was last revised and confirmed in 2020. The photocatalytic self-cleaning activity is measured through the hydrophilicity of the self-cleaning material after being fouled with oleic acid and irradiated with UVA light. More details on the protocol are stated in Section 2. In this study, it is our intention to use the ISO 27448:2009 standard to compare the photocatalytic self-cleaning activity of a variety of industrial self-cleaning surfaces, hygienic materials and materials with an experimental self-cleaning coating. By doing so, we will reveal the benefits and the (unfortunately many) limitations of the standard, highlight the importance of data interpretation, and provide alternative testing procedures.

## 2. Materials and Methods

**Figure 2:**
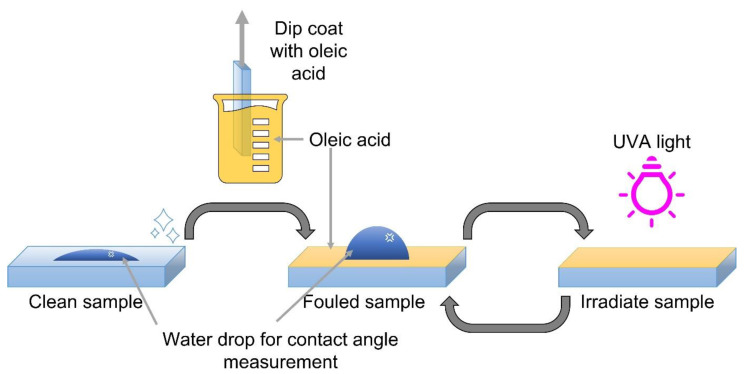
Experimental scheme of ISO 27448:2009 where the water contact angle of a clean sample is measured, the sample is fouled with oleic acid via dip coating, measured again for its water contact angle and irradiated with UV-A light alternatively until the final angle is reached.

All samples were tested according to ISO 27448:2009 Test method for self-cleaning performance of semiconducting photocatalytic materials—Measurement of water contact angle [31]. The method describes the testing of five identical samples. Square-shaped samples of 50 mm by 50 mm were used. After a pre-treatment under UVA light for 24 h at an intensity of E = 2.0 mW cm^−2^ to eliminate traces of organic fouling from the surface, the samples were fouled with oleic acid using the dip coating method. The samples were submerged in 0.5 v% oleic acid (Sigma-Aldrich, USA, MI, Saint Louis, 90%) in n-heptane (Chem-Lab NV, 99+ %) and withdrawn at a withdrawal speed of 60 cm min^−1^. After drying them for 15 min in an oven of 70 °C and cooling them down to room temperature (23 ± 5) °C, the samples were ready for testing. The samples were irradiated using a Philips fluorescence S 25W UV-A lamp, E = 2 mW cm^−2^ for wavelengths λ < 400 nm, ad measured using a calibrated spectroradiometer (Avantes Avaspec-3648-USB2). At certain time intervals (t), five water droplets of 4 µL for each sample were measured using a contact angle goniometer (Ossila) starting at t = 0 min, before the irradiation. For each droplet, a video of 10 s at 5 frames per second was captured from the moment right before the droplet touched the sample surface, and it was analysed using the Ossila contact angle software. The equilibrated water contact angle of each droplet was calculated from the last ten frames of each video. The mathematical average of the last three measuring points with a relative error of lower than 10% was defined as the final contact angle (θ_f_). A simplified scheme of the experimental set up is given in Figure 2. The samples tested in this study made up a wide variety of materials: kitchen countertops (further denoted as ‘Surface materials’ A and B), kerbs (samples ‘SaniCoat’ and ‘CleanRock’ produced by PolySto), titania-treated carpet tiles and luxury vinyl tiles (LVT) (denoted as ‘Carpet tile ref’, ‘Carpet tile treated’, ‘LVT ref’ and ‘LVT treated’), roofing material (‘Roofing material’), architectural panels (‘Architectural panel’ A and B) and a self-cleaning coating developed by our research group applied on glass (‘DuEL TiO_2_’) and on architectural panels (‘DuEL Architectural panels’ A and B). The in-house developed self-cleaning coating was patented (PCT/EP2018/079983) [32], and its specifications have been described in detail elsewhere [3]. The commercial materials listed above were provided by a variety of companies from different industries. Since these materials are either still under development, and/or are patented by the respective companies, we are not allowed to report all of the company names, nor are we allowed to reveal the basic characterization data of the samples, other than the experimental results of the ISO test, due to legal and confidentiality issues. Therefore, most of the materials are labelled with a generic name, as summarised in Table 1.

## 3. Results

**Figure 3:**
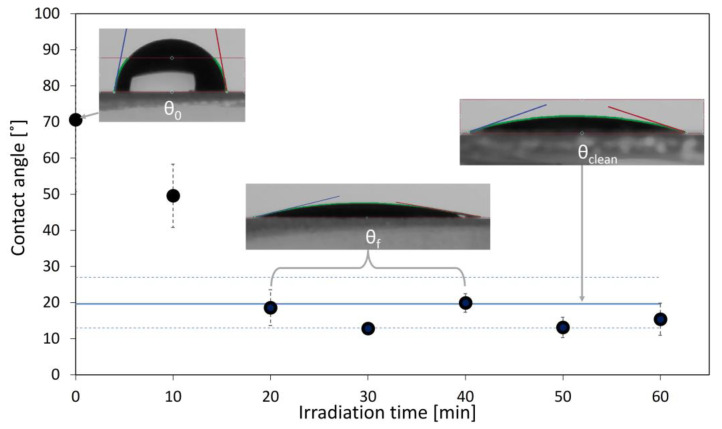
Example of the typical evolution of the water contact angle for a smooth, hydrophilic, photocatalytic self-cleaning surface, in this case, it is DuEL TiO_2_. The blue line is the clean surface angle with blue dashed lines as error bars. The black dots show the degradation of the deposited oleic acid as the surface regains its hydrophilicity over time.

As mentioned above, since most of the samples are confidential industrial materials, no characterisation other than the water contact angle measurements were allowed to be performed. The only exception is the in-house developed titania coating (DuEL) on Borofloat^®^ glass, which was fully characterised in our previous study [3]. An example of the evolution of the water contact angle of a typical smooth, hydrophilic, photocatalytic self-cleaning surface is given in Figure 3, with images of a water droplet for the clean, pre-treated, unfouled surface (θ_clean_), the fouled surface with oleic acid before UV irradiation (θ_0_) and the final contact angle after UV irradiation (θ_f_).

**Figure 4:**
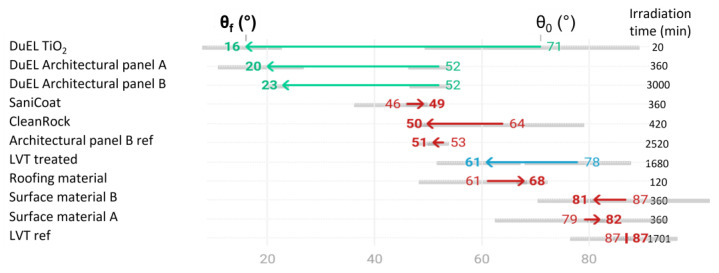
Overview of the evolution the water contact angle from the oleic-acid-fouled surface before UV irradiation (θ_0_) to the final contact angle after UV irradiation (θ_f_) and the corresponding required irradiation time (t_f_) in minutes. The samples can be divided into three groups: the photocatalytically active samples (green arrows), the semi-active samples (blue arrows) and the inactive samples (red arrows). The grey bars represent the experimental error.

An overview of the water contact angles of the clean, pre-treated, unfouled surface (θ_clean_), the fouled surface with oleic acid before UV irradiation (θ_0_) and the final contact angle after UV irradiation (θ_f_) and irradiation time that needed to be reached θ_f_ (t_f_) are given in Table 1. A dash (-) in the table means that the sample could not be measured according to the guidelines specified by the standard. These cases are discussed in more detail below. Figure 4 shows the evolution from θ_0_ to θ_f_, and it also summarises t_f_ for all of the samples.

It should be clear that the clean contact angle varies greatly depending on the material’s surface. The more synthetic materials show rather hydrophobic angles, whilst the samples treated with the DuEL coating (DuEL and DuEL Architectural panels A and B) are characterised by hydrophilic to superhydrophilic angles. The contact angle logically depends on the surface chemistry (e.g., coating) of the investigated material. Yet, there is also a clear noticeable influence of the underlying substrate, as evidenced by the different clean contact angles of DuEL Architectural panel A and B samples and the DuEL TiO_2_ sample. 

In Figure 3, the samples are arranged by their final angles. First, a very important point that needs to be stressed here is that a large final angle does not necessarily imply that the sample is not photocatalytically active, since the water contact angle also strongly depends on the hydrophilicity or -phobicity of the original sample’s surface and not only on the remaining amount of oleic acid. Hence, a photocatalytically active surface of a synthetic (hydrophobic) substrate will thus show a larger, hydrophobic, clean and final water contact angle, allowing it to appear that little to no oleic acid has been removed, while in practice, it can simply not be measured. Based on the results of the ISO standard test, the samples can be divided into three groups: the photocatalytically active samples (green), the semi-active samples (blue) and the (seemingly) inactive samples (red). This arbitrary division is based on the difference in the water contact angles before and after irradiation, θ_0_ and θ_f_, respectively. When a substantial difference is found (θ_0_/θ_f_ > 2), the sample is categorised as active. When the difference is less significant, but the final angle is still lower than the initial angle and not within the range of error of the measurement (θ_0_/θ_f_ > 1 and θ_0_/θ_f_ < 2 with θ_f_ < (θ_0_—standard deviation [sd])), the sample is categorised as semi-active. When the difference is insignificant (θ_0_/θ_f_ = 1), the final angle is larger than the initial angle (θ_0_/θ_f_ < 1) or the final angle falls within the error margin of the initial angle (θ_f_ > (θ_0_—sd)), the sample is categorised as photocatalytically inactive according to this standard. The three samples categorised as active all have the patented titanium dioxide coating by DuEL. The difference in the photocatalytic activity between these samples might not actually be visible when we are looking at the final angle. Only when we are also comparing the irradiation times needed to reach these final angles, a clearer difference can be seen. The DuEL coating on the Borofloat^®^ glass (DuEL TiO_2_) required only 20 min to reach the final angle, whereas the DuEL-treated samples, DuEL Architectural panels A and B, required 360 min and 3000 min, respectively, thus the times are one and two orders of magnitude longer. Out of the other samples, only the LVT sample treated with titanium dioxide shows photocatalytic activity, but not to the same extent as the previously mentioned samples. These differences might be due to the influence of the substrate or certain steps in the fabrication process that may have had adverse effects on the photocatalytic properties, e.g., thermal polarisation effects upon heat treatment or the doping of the coating by contaminants from the substrate, leading to excessive charge recombination. Despite the smaller final angles of the SaniCoat and CleanRock samples and the Architectural panel B ref sample compared to that of the treated LVT sample, these samples do not show any photocatalytic activity, as θ_f_ is within the margin of uncertainty, or it is even larger than θ_0_, as is the case for the samples with larger final angles: the Roofing material, the Surface materials A and B and the LVT reference, which can seen on the bottom halves of Table 1 and Figure 4. The samples at the very bottom of the table are the samples that could not be measured, which are represented here with a dash.

Only a few accounts in the literature report on test results obtained with the ISO 27448:2009 method. TiO_2_ sol-gel-coated industrial ceramic tiles required 25 or 50 h reach a final angle of below 20° at 2 mW cm^−2^ using the same light intensity as that which was used in this research [33]. Commercial glazed ceramic tiles functionalised with a micrometric TiO_2_ layer required 77 h to approach their clean angle of 12.6° at half of the light intensity as that which we used [34]. Transparent TiO_2_ and ZnO thin films for polymeric sheets even needed 120 and 180 h of irradiation, respectively, to achieve a final angle of below 5° at 1 mW cm^−2^ [35]. In contrast, the most active DuEL-coated sample in the present study only required 20 min of irradiation to reach the final angle of (16 ± 4)°. Even the example given in the standard protocol itself reports an irradiation time of 70 h before the final angle is reached, clearly confirming the superior performance of our DuEL-coating as a smooth, hydrophilic self-cleaning photocatalytic layer.

## 4. Discussion

**Figure 5:**
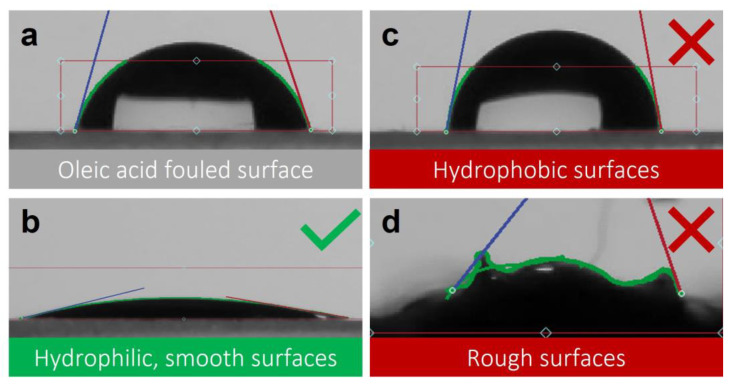
Water droplets on top of different surfaces: a surface fouled with oleic acid (**a**), a clean, smooth and hydrophilic surface (**b**), a clean and hydrophobic surface (**c**) and a clean and rough surface (**d**). For rough surfaces, the water contact angle cannot be measured, and for the hydrophobic surface, the standard cannot be used, which is indicated by the red color and crosses. The green color and checks indicate that hydrophilic, smooth surfaces are suitable for this standard.

A first and very important observation is that the ISO 27448:2009 method is not applicable to any given sample. Firstly, the method is unsuited to test samples with a very high surface roughness value, as illustrated in Figure 5. Hence, the carpet tiles with a bristle-like surface could simply not be measured using this ISO test, as no water droplets are formed on top of the bristles. Secondly, other samples that are excluded by this standard include water-permeable substrates, highly hydrophobic, powderous or granular materials and visible light-sensitive photocatalysts [31]. For the hydrophobic samples with a contact angle of the clean, pre-treated, unfouled surface (θ_clean_) that is larger than the initial contact angle (θ_0_ after fouling and before UV irradiation), this test will always be inconclusive. In this case, the degradation of oleic acid cannot be measured by the recuperation of hydrophilicity of the material, since the material is not hydrophilic to start with, or at least it is less hydrophilic than the fouled material, which is also visualised in Figure 5. The other disadvantages of this standard are the exclusion of visible light active photocatalysts, and the free choice between the two different methods for applying the oleic acid layer (dip coating versus manual application). Each method comes with a different required irradiance, but without any explication on how or why to choose a given method, which was also pointed out by Mills and Banerjee [36,37]. Another difference between both of the methods of application is that for the dip coating method, there is no intension to quantify the applied amount of oleic acid. The standard does not prescribe measuring a reference sample (e.g., a non-active sample (plain glass) fouled with oleic acid and irradiated in the same way) to account for the possible photodegradation of the oleic acid. Additionally, the volume of the droplet for the water contact angle analysis is not defined, which accounts for that the fact that different articles report different droplet volumes over a range of a few µL. Despite these drawbacks, the standard also has some benefits. Besides the benefits of any standardised protocol to accurately compare the samples under the same conditions, the main benefit of ISO 27448:2009 is its simplicity. The equipment needed for this standard is also not very expensive. An automatic goniometer setup is convenient, and it comes in different price ranges, but nowadays, even smartphone cameras are of high enough quality to take an accurate picture, which can be analysed using freely available software.

We need to stress again here that the fact that because some samples cannot be evaluated using this standard, it does not mean that they are not photocatalytically active. An alternative test for the photocatalytic activity can be performed instead. When we are looking at the available ISO standards, ISO 10678:2010, which tests the photocatalytic activity in an aqueous medium by the degradation of methylene blue, could provide a first alternative. For this test, the initial water contact angle does not matter, and thus, more hydrophobic samples can be measured as well. However, given the high adsorption capacity of the samples with high surface roughness values, this standard does not seem to be the best option for, e.g., the carpet tile samples. The high adsorption capacity can lead to an apparent degradation of methylene blue since this compound is removed from the solution by adsorption onto the surface of the material rather than being degraded photocatalytically. To prevent this, an adsorption period is prescribed by the standard. However, given the extremely high adsorption capacity of the carpet tiles, the adsorption phase would become so long, that the test is no longer practical. Another ISO test that could be performed is ISO 22197:2016 for photocatalytic air purification by the removal of different gaseous compounds. It is important to mention that these standards can be used to indicate the photocatalytic activity of a material, but they are not suited to measure the photocatalytic self-cleaning activity. In the mid-1990s of the previous century, a different method for the evaluation of the photocatalytic self-cleaning activity of glass was proposed by Paz and colleagues [38]. In this method, a thin film of stearic acid, a model compound for organic fouling that is rather similar to oleic acid used in ISO27448, is spin coated onto the self-cleaning samples. Using Fourier Transform Infrared spectroscopy (FTIR), the photoactivity of the samples is calculated by the rate of decrease in the integrated absorbance of C-H stretching vibrations between 2700 and 3000 cm^−1^. An important condition for this testing method, is that the test samples need to be IR transparent, which unfortunately excludes most of the commercial applications. More recently, another alternative test method has been proposed using smart inks to test the photocatalytic activity of surfaces. In 2009, when ISO 10678:2010, which is used to assess the photocatalytic activity in aqueous media by methylene blue degradation, was under development, Zita, Krýsa and Mills cooperated on the development of an alternative testing method for photocatalytic films. They employed an ink containing the redox dye resazurin as a smart ink that changes color when it is exposed to a photocatalytic reaction. Rather than degrading the dye such as in the case of methylene blue, resazurin changes the color from blue to pink by titania-sensitised reduction. Later on, two more photocatalytic activity indicator inks were introduced: Basic Blue 66 (from blue to colorless) and Acid Violet 7 (from pink to colorless). Given the strong correlation in the photooxidation rates between methylene blue and resazurin oxidation and the high repeatability and reproducibility on top of the faster (less than 10 min), easier and cheaper use of the smart ink test, this (semi-)quantitative method shows great potential. It could boost rapid quality assurance, correct fitting, marketing and in situ assessment to identify ‘fake’ or photocatalytically inactive products quickly [39]. The method was developed for self-cleaning glass, but also, photocatalytic paints and tiles have been tested [8,40,41]. Since then, several publications have made successful use of this method to measure the photocatalytic activity of self-cleaning materials [39,40,42,43,44,45]. In 2018, ISO adapted this method, establishing a new standard ISO 21066:2018 [46].

**Figure 6:**
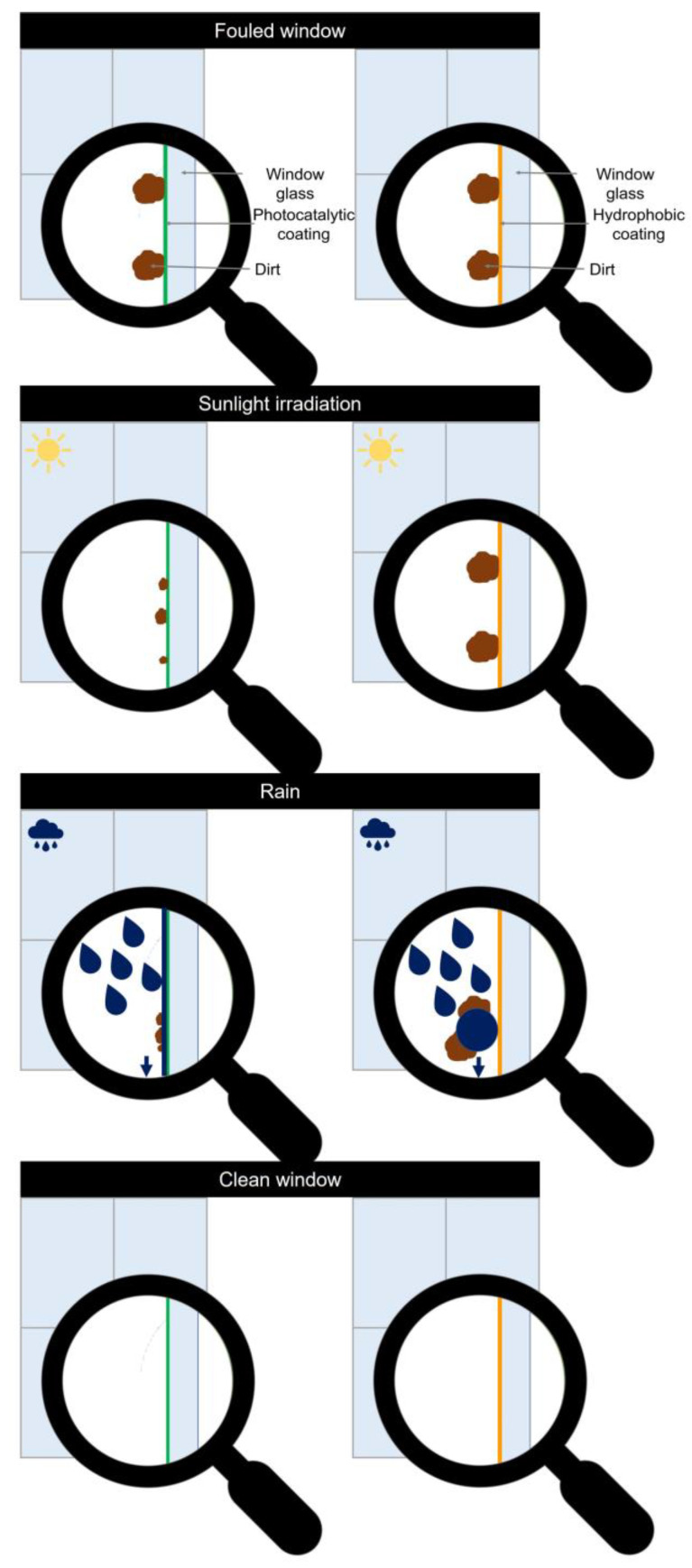
The self-cleaning mechanisms for a photocatalytic, hydrophilic coating on the left and a superhydrophobic coating on the right.

A second important remark is that a surface that does not show any photocatalytic activity is also not necessarily not self-cleaning. As a matter of fact, there are two main strategies to produce a self-cleaning surface. Either one can make the surface photocatalytically active and hydrophilic, as the materials suitable for the performed ISO standard- or render the surface (super)hydrophobic [2]. The latter method makes use of the water-repelling effect of (super)hydrophobic surfaces, where the formed water beads trap the foulants and carry them off the surface. For this, an inclination angle of at least 10° from the horizontal plane is needed. On top of this, it is harder for the pollutants to stick to the material since the polymers and resins used to make the surface hydrophobic smoothen out the microscopically pitted and pocked surface [2]. A material is categorised as hydrophobic when it has a water contact angle of typically 104° or greater. To be categorised as a hydrophilic material, the water contact angle needs to be below 90°. The different self-cleaning mechanisms are illustrated in Figure 6.

It is equally important to realise that a material that is not self-cleaning is therefore not, by definition, unhygienic. According to a review by Midtdal and Jelle, most of the hydrophobic materials mentioned above actually show a poorer self-cleaning action than the uncoated float glass does. Real-life conditions of outdoor hydrophobic self-cleaning glass hardly ever provide the necessary high impact pressure of water droplets (rain) needed to evacuate the fouling particles. Yet, the hydrophobic coating can reduce and help the cleaning process of the material, since the smoothened surfaces make it harder for foulants to attach to it. These materials can thus be labelled as ‘easy-to-clean’, rather than ‘self-cleaning’ [2]. More recently, also, superhydrophobic coatings based on the self-cleaning properties of the Lotus leaf, ‘the Lotus Effect’, have been developed. To be categorised as superhydrophobic, a surface needs a water contact angle of 150° or more and a roll-off angle of less than 10° [47,48]. Here, rough surface structures and very low surface energies, which are often achieved by silane-based surface modifications, make it hard for the contaminants to adhere to the surface. Instead, the foulants will adhere to water droplets and roll off the surface together. More specifically, the stronger capillary effect compared to the adhesion effect enables contaminant removal. However, water, mostly in the form of rain, is necessary to obtain the self-cleaning activity, as well as the ‘easy-to-clean’ property. These surfaces can also show anti-icing, anti-corrosion, drag reduction, anti-biofouling, antifogging, self-healing, UV and thermal resistance properties [47,48]. Superhydrophobic self-cleaning surfaces have spread to applications far beyond construction materials, and they are also employed, for example, in display devices [7]. Special attention should be given to the durability of hydrophobic coatings, especially when they are applied by the customer themselves in DIY kits. A lifetime of between 3 and 4 years is expected, whereas a lifetime of 10 years is promised by some producers when the coating is applied by professionals. Photocatalytic self-cleaning coatings are usually applied during the production process, and they are fused with the material, giving them an expected lifetime of up to 30 years for self-cleaning glass, which is the same lifetime as the substrate, e.g., a window itself [2].

## 5. Conclusions

When one is performing ISO 27448:2009 on a variety of industrial samples and an in-house developed titania coating (DuEL) both on Borofloat^®^ glass and architectural panels, only the DuEL-coated samples show actual photocatalytic self-cleaning activity, according to this standard. The Roofing material treated with titania shows some photocatalytic self-cleaning activity, but the final water contact angle after fouling and irradiation remains large. All of the other tested materials failed to show any activity according to the standard because their surface was either too rough or too hydrophobic. It is our conclusion that ISO 27448:2009 is not necessarily the best test to evaluate the photocatalytic self-cleaning activity of materials. Only a very specific subset of materials is suitable for this test, namely non-powdery, non-granular, hydrophilic materials with surfaces that are smooth enough to support water droplets and the ability for us to perform reliable water contact angle measurements as described by the standard. For substrates that are not hydrophilic, a different test for measuring the photocatalytic activity should be performed. A good candidate test would be the color change measurement using smart inks such as resazurin, Acid Violet 7 or Basic Blue 66. This test can be performed on various substrates and under various conditions, whereas the standard for methylene blue degradation (ISO 10678:2010) evaluates the photocatalytic activity in aqueous media. The proposed method using smart inks is also faster, easier and cheaper for testing the photocatalytic activity than the current ISO standards that are mentioned above are. On the other hand, is it important to mention that a surface that is not photocatalytically active according to the standard is therefore not self-cleaning or unhygienic. While there used to be a lack of proof of the self-cleaning activity of hydrophobic surfaces, recent research into superhydrophobic surfaces might actually achieve a non-photocatalytic self-cleaning function besides self-healing, anti-icing, antibiofouling, anti-corrosion, drag reduction, anti-fogging, UV and thermal and chemical resistance properties, which call the attention of various industries. The previously mentioned hydrophobic surfaces can be labelled as ‘easy-to-clean’, and they can reduce the frequency of cleaning procedures. For the complete degradation of organic contaminants, photocatalytic self-cleaning coatings are, for now, still the best option, since these coatings actually mineralise the foulants instead of only rinsing them off upon exposure to water. 

## Figures and Tables

**Table 1:** Overview of the water contact angles of the clean, pre-treated, unfouled surface (θ_clean_), the oleic-acid-fouled surface before UV irradiation (θ_0_), the final contact angle after UV irradiation (θ_f_) and irradiation time needed. A dash (-) is used for values that could not be measured.

Sample	θ_clean_ (°)			θ_0_ (°)			θ_f_ (°)			t_f_ (min)
DuEL TiO_2_	20	±	7	71	±	20	16	±	4	20
DuEL Architectural panel A	5	±	4	52	±	2	20	±	4	360
DuEL Architectural panel B	3	±	2	52	±	2	23	±	1	3000
SaniCoat	88	±	5	46	±	4	49	±	1	360
CleanRock	71	±	17	64	±	16	50	±	1	420
Architectural panel B	43	±	6	53	±	1	51	±	1	2520
LVT treated	70	±	13	78	±	5	61	±	8	1680
Roofing material	57	±	3	61	±	6	67.7	±	0.5	120
Surface material B	78	±	8	87	±	8	81.0	±	0.4	360
Surface material A	77	±	7	79	±	8	82	±	2	360
LVT ref	93	±	7	87	±	5	87	±	1	1710
Carpet tile treated	-	±	-	-	±	-	-	±	-	-
Carpet tile ref	-	±	-	-	±	-	-	±	-	-

## Data Availability

The data presented in this study are available on reasonable request from the corresponding author.
